# The Importance of Material Used in Speech Therapy: Two Case Studies in Minimally Conscious State Patients

**DOI:** 10.3390/brainsci12040483

**Published:** 2022-04-08

**Authors:** Alice Sautet, Laura Hurtado, Anna Fiveash, Leslie Baron, Mélaine De Quelen, Fabien Perrin

**Affiliations:** 1 CAP Team (Cognition Auditive et Psychoacoustique), Lyon Neuroscience Research Centre (Université Claude Bernard Lyon 1, INSERM U1028, CNRS UMR5292), 69675 Bron, France; alicesautet@gmail.com (A.S.); hurtado.laura34@gmail.com (L.H.); anna.fiveash@inserm.fr (A.F.); 2 Speech Therapy Department, Université de Nantes, 44035 Nantes, France; leslie.baron@hotmail.fr; 3 Neuro-Rehabilitation Unit, Neurological Hospital Pierre-Wertheimer, Hospices Civils de Lyon, 69677 Bron, France; melaine.de-quelen@chu-lyon.fr

**Keywords:** music, autobiographical memory, tempo, speech therapy, minimally conscious state

## Abstract

Speech therapy can be part of the care pathway for patients recovering from comas and presenting a disorder of consciousness (DOC). Although there are no official recommendations for speech therapy follow-up, neuroscientific studies suggest that relevant stimuli may have beneficial effects on the behavioral assessment of patients with a DOC. In two case studies, we longitudinally measured (from 4 to 6 weeks) the behavior (observed in a speech therapy session or using items from the Coma Recovery Scale—Revised) of two patients in a minimally conscious state (MCS) when presenting music and/or autobiographical materials. The results highlight the importance of using relevant material during a speech therapy session and suggest that a musical context with a fast tempo could improve behavior evaluation compared to noise. This work supports the importance of adapted speech therapy for MCS patients and encourages larger studies to confirm these initial observations.

## 1. Introduction

Following a coma, severely brain damaged patients may progress to a disorder of consciousness (DOC). The vegetative stage/unresponsive wakefulness syndrome (VS/UWS) is characterized by the presence of eye-opening periods and reflex movements, without signs of awareness [1]. The minimally conscious state (MCS) refers to patients with fluctuating but reproducible signs of self- and environmental awareness [2]. The complexity of their conscious behaviors distinguishes patients in MCSs. MCS- is characterized by low-level behaviors, such as visual pursuit, pain localization, and affective behaviors appropriate to the emotional context, and MCS+ refers to patients with higher-level behaviors, such as responses to a motor command, yes/no coding, and nonfunctional but intentional communication [3]. Emergence from an MCS is suggested when the patient can appropriately use at least two objects of daily living and/or has functional communication [4].

Patients with a DOC require daily care by a multidisciplinary team, including speech therapists. The latter are involved in the evaluation and rehabilitation of swallowing, phonation, communication, and language functions [5]. The evaluation of swallowing is an important part of a speech–language pathologist’s practice, as dysphagia has major functional consequences and comorbidities. Several scales are used to assess swallowing disorders, such as the Facial Oral Tract Therapy Swallowing Assessment of Saliva [6] and, very recently, the Swallowing Assessment in Disorders of Consciousness [7]. In daily practice, a speech–language pathologist also presents multisensory stimuli, including words, to encourage verbalization, which allows him/her to assess language functions (comprehension and production). On the basis of these evaluations, a speech–language pathologist can propose rehabilitations, for example, a multisensory stimulation (visual, auditory, tactile, olfactory, and gustatory) program over several weeks (e.g., [8,9,10]). In this context, a speech therapist favors pleasant stimuli that are in line with the patient’s previous tastes to reactivate affective memories and stimulate episodic memory [11]. However, while a speech–language pathologist’s tasks with DOC patients are well-defined, there is limited research and official recommendations for the content of speech therapy sessions.

Neuroscientific research on the cognitive processing abilities of DOC patients suggests that personally relevant stimuli (i.e., with emotional, autobiographical, or self-related characteristics) increase the chance of observing behavioral or brain responses compared to neutral stimuli [12]. For example, their mother’s voice [13], familiar faces [14], short sentences spoken by a family member [15], or the patient’s own first name [16] increase the chances of observing a brain response compared to a neutral stimulus. This research also shows that behavioral and brain assessments are more frequently observed if they are preceded by a personally relevant context compared to a neutral context [12]. Indeed, Verger et al. and Heine et al. showed the beneficial effect of a patient’s favorite music on their behavioral performance, assessed with items from the Coma Recovery Scale—Revised (CRS-R), as compared to a noise control condition [17,18]. Similarly, the use of a patient’s preferred odor improved performance on the CRS-R as compared to a neutral odor, but with a lower effect than that observed for a preferred music context [18]. Furthermore, a study by Castro et al. found that the likelihood of observing a cerebral response to the patient’s own first name increased twofold after a preferred music context compared to a neutral noise context [19].

Among the stimuli used in previous research, music appears to be an especially relevant stimulus. Indeed, listening to music has been proven to be a powerful tool to investigate brain plasticity, as it involves a large number of cortical and subcortical brain regions (for a review, see [20]), and influences emotional, sensory–motor, and cognitive processing (e.g., [21,22,23,24]). Music listening could have both short-term and long-term effects in DOC patients. In the short-term, it could increase the sensitivity of the evaluation, and in the long-term it could improve the effect of rehabilitation programs (for a review, see [25]). For example, some studies suggest that it could be a useful clinical tool in stimulating a range of behavioral, physiological, and expressive responses in patients in low-awareness states [26,27]. In particular, music listening could improve expressive communication in cases of neuro-communication disorders [28,29,30,31,32], and could help to better diagnose patients by improving the sensitivity of tests [19]. This latter short-term beneficial effect could be explained by increased connectivity in brain regions involved in consciousness, language, rhythm, emotion, and autobiographical memory processing [33,34]. However, while it has been suggested that the preference (its familiarity, emotional, and autobiographical characteristics) of the music contributes to the beneficial effects [18], it is still unclear if acoustic features also influence these effects for DOC patients. Among the different acoustic features of the music, its tempo could be considered as an important generator of physiological responses. Indeed, an increase in the tempo has been shown to increase heart rate and blood flow in the brain [35,36]. A fast tempo appears to increase the activity of the sympathetic nervous system, thus emotional and physiological arousal [35], while a slow tempo appears to influence parasympathetic nervous activity, responsible for relaxation [37].

Taken together, these studies suggest that speech therapy sessions in DOC patients could be improved by using autobiographical stimuli and/or music. We have investigated this issue using two complementary approaches. On the one hand, we have investigated the effect of an autobiographical speech therapy session compared to that of a neutral, standardized speech therapy session (as currently used in practice) on behavior within the session. On the other hand, we have evaluated the effects of a musical context, and its tempo, on a subsequent behavioral assessment as compared to silent or noise contexts, respectively. Among DOC patients, we chose two patients in MCSs to ensure more variable behavioral responses. We also chose to conduct case studies to investigate effects at the individual level. By applying the same protocol from 4 to 6 weeks, we were able to repeat the conditions several times to increase the power of the individual results. Although the results of these case studies cannot be generalized to larger populations, they allow for an important first step in the investigation of material types within speech therapy sessions for patients in MCSs. It is particularly difficult to obtain generalizable results with a group of MCS patients, given the variability of their states. Therefore, individual case studies conducted over multiple sessions provide valuable insights into quantitative and qualitative changes in behavior.

Case study one aimed to evaluate, in an MCS patient, whether autobiographical, multisensory speech therapy sessions could enhance oromotor/verbal and communication skills within the sessions, compared to standard neutral speech therapy sessions. In order to avoid interfering with the work of the speech therapist during the session, we did not use a standard scale but we rather quantified, as objectively as possible, the behavioral responses to the presentation of three autobiographical (autobiographical sessions) or neutral (neutral sessions) multisensory stimuli proposed by the speech therapist. A list of eight behaviors were defined to consider (1) oral and perioral abilities, (2) premises of communication during social interaction, and (3) communication skills, i.e., the measures that help speech therapists to assess improvement from an MCS. A second aim of this case study was to evaluate the effect of a preferred music context on the subsequent speech therapy session, compared to a silent context. While neuroscientific studies suggest that a music context improves subsequent behavior compared to a noise context, we wanted to know if this was also the case compared to a silent context.

Case study two aimed to evaluate, in an MCS patient, whether a fast-tempo context (fast music) could improve the subsequent behavioral assessment compared to slow-tempo (slow music) and noise contexts. In this case study we chose to follow previous neuroscientific studies as closely as possible by using noise as a control condition and testing performance on CRS-R items (as in [17,18]), in order to isolate specifically the effects of tempo.

## 2. The Effect of Autobiographical Material during a Speech Therapy Session. Case Study One

### 2.1. Method

#### 2.1.1. Patient

Mrs. D., 41 years old, was hospitalized following a subarachnoid hemorrhage that resulted in a left temporal and subdural hematoma (traumatic hemorrhagic brain injury). After a coma phase (Glasgow Coma Scale: 3), she evolved towards an MCS. She had right hemi neglect and mixed aphasia. The present experiment started 30 days after the onset of coma. At the beginning of the present study, the patient was stable, nonsedated, had no indication of deafness (i.e., brainstem auditory-evoked potentials were present), and was in an MCS- (13 points on the CRS-R: auditory function = 2 points, visual function = 3 points, motor function = 5 points, oromotor/verbal function = 1 point, communication = 0 points, and arousal = 2 points). At the end of the present study, the patient was still in an MCS- (CRS-R: 13 points). However, caregivers reported more stereotypies, yes/no responses, and communicative gestures.

The examinations took place in the intensive care unit and then in the post-intensive care rehabilitation unit of the Pierre Wertheimer neurological hospital in Bron (France). The experiment was conducted in agreement with the guidelines of the Declaration of Helsinki. In accordance with French legislation at the time of the study, a written information form was given to the patient’s relative.

#### 2.1.2. Materials

A questionnaire for families was created to select autobiographical material associated with episodic and semantic memories. The patient’s perfume (olfactory stimuli), favorite desserts (olfactory or gustatory stimuli), personal photos (visual stimuli), letters recorded by the family and read out (auditory stimuli), and familiar objects brought by the family (tactile stimuli) were selected to be used during the “autobiographical speech therapy session” (aSP, see below). We also asked family members to indicate five of the patient’s preferred pieces of music (to use before the speech therapy session, i.e., as a preferred music context, mC, see below).

Neutral stimuli, to be used during the “neutral speech therapy session” (nSP, see below), were also selected: neutral desserts, such as as plain yoghurt (olfactory or gustatory stimuli), neutral photos from ColorCards [28] (visual stimuli), recordings of everyday sounds such as crowds (auditory stimuli), and objects without personal connotations (tactile stimuli).

#### 2.1.3. Experimental Protocol

**Figure 1:**
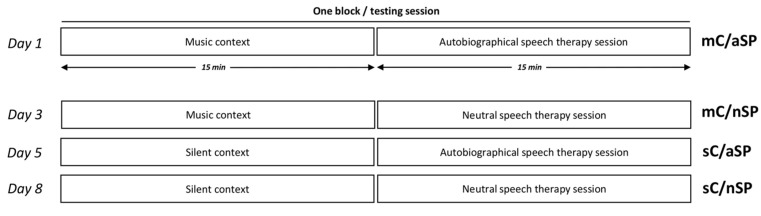
Experimental design for case study one. The four types of blocks (a preferred music context followed by an autobiographical speech therapy session, mC/aSP, a preferred music context followed by a neutral speech therapy session, mC/nSP, a silent context followed by an autobiographical speech therapy session, sC/aSP, and a silent context followed by a neutral speech therapy session, sC/nSP) were pseudo-randomly presented 3 times each throughout 4 weeks. The first four blocks are illustrated, corresponding to days 1, 3, 5 and 8.

The study was conducted over four consecutive weeks, and consisted of 12 blocks of 30 min sessions (three blocks per week for four weeks, two or three days apart, the same days/hour each week). One block (i.e., one testing session) consisted of a 15 min context (C) followed immediately by a 15 min speech therapy session (SP) (Figure 1).

Four types of blocks were pseudo-randomly presented three times (a homogeneous distribution of the four conditions was respected over time): (a) a preferred music context followed by an autobiographical speech therapy session (mC/aSP), (b) a preferred music context followed by a neutral speech therapy session (mC/nSP), (c) a silent context followed by an autobiographical speech therapy session (sC/aSP), and (d) a silent context followed by a neutral speech therapy session (sC/nSP).

The preferred music context (mC) was made up of two to three favorite music pieces (the 5 selected pieces were distributed randomly between the blocks) delivered by speakers placed 1.5 m away from the patient. Earplugs were inserted for the silent context (sC).

#### 2.1.4. Behavioral Assessment

Speech therapy sessions (SPs) consisted of soliciting the two chemical modalities (olfactory and gustatory) and one of the three other sensory modalities (visual, auditory, or tactile). Both olfactory and gustatory stimuli were proposed in each session to allow the reafferentation of the oral sphere (for approximately 10 min). Photos were presented (visual modality), recorded sounds were played (auditory modality), and objects were put to the skin (tactile modality) for approximately 5 min. Autobiographical speech therapy sessions (aSPs) and neutral speech therapy sessions (nSPs) only differed in the nature of the stimuli. The number of sensory modalities per session was limited to three (olfactory/taste/touch; olfactory/taste/visual; and olfactory/taste/auditory) to avoid the presentation of too many stimuli and the attentional saturation of the patient. Thus, the speech therapy session could encourage and work on: (1) oral and perioral reafferentation via chemical stimuli; (2) the emergence of communication, access to the lexicon, and lexical oral comprehension (naming) via visual stimuli; (3) verbal/nonverbal communication and oral comprehension via auditory stimuli; and (4) oral comprehension (simple commands), verbal/nonverbal communication, and ideatory praxis via tactile stimuli.

The aim of the present case study was to measure the effects of a combination of stimuli on the patient’s behavior within the speech therapy sessions. Thus, the objective was not to validate a new scale but to evaluate the most pertinent behaviors relevant for speech therapists. To quantify the behavioral responses to the presentation of the 3 autobiographical (aSP) or neutral (nSP) multisensory stimuli by the speech therapist, we predefined a list of the 8 most pertinent behaviors for speech therapists. It took into account oral and perioral abilities, premises of communication during social interaction (eye contact, attention, and emotional expression), and communication skills (responses to simple commands, yes/no code, vocal or verbal reactions, and motor manifestations). Each of these behaviors help speech therapists to assess improvement. The list of behaviors was selected from different validated scales (the CRS-R [38], the Wessex Head Injury Matrix [39], and the Communication Assessment of Patients in the Awakening Phase of Coma [40]), as well as a speech–language pathology assessment for brain-damaged patients, the Test Lillois of Communication [41]. Each of the 8 behaviors was scored based on a hierarchy of the appearance of the behaviors, from the absence of manifestation or the presence of a reflex behavior to the most complex behavior (from 0 to 2 or 0 to 3 points). We additionally included a qualitative assessment (see Table A1).

#### 2.1.5. Data Analysis

Behavioral assessments for the 12 speech therapy sessions were completed by two independent speech therapists who were blind to the condition (scoring was conducted from videos that were cut to disguise the experimental context). The inter-rater reliability was very high (95.19%), so the average scores of the two evaluators were used for the data analysis. The scores were normalized to each other (as the maximum scores were 2 or 3) and expressed as a percentage of the maximum possible score. The data were qualitatively presented according to the four conditions (mC/aSP, mC/nSP, sC/aSP, and sC/nSP) and to the eight behaviors.

As previously done in single-case analyses (e.g., [18,42]), statistical analyses were performed on the average scores of the 8 behaviors in each speech therapy session, using nonparametric tests (Friedman and Wilcoxon tests), and reported when significant (*p* < 0.05).

In order to observe a potential evolution of the scores over time, they were also averaged for each week, regardless of condition. As scores increased linearly over the four weeks, the linear regression curve was subtracted from each of the average scores (to remove a possible spontaneous recovery). Statistical analyses were also performed on these scores using nonparametric tests (Friedman and Wilcoxon tests) and reported when significant (*p* < 0.05).

### 2.2. Results

**Figure 2:**
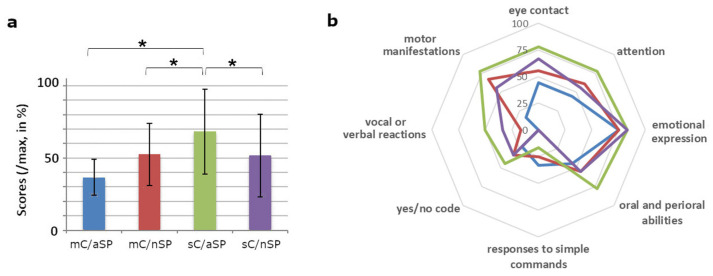
Behavioral scores to mC/aSP (musical context/autobiographical speech therapy session, blue), mC/nSP (musical context/neutral session, red), sC/aSP (silent context/autobiographical session, green), and sC/nSP (silent context/neutral session, purple). (**a**) The average scores were expressed according to the 4 conditions (“/max, in %” means percentage of the maximum possible score, * means *p* < 0.05). (**b**) The average scores were expressed according to the 4 conditions and to each of the 8 behaviors (percentage of the maximum possible score).

The average scores were expressed according to the four experimental conditions (Figure 2a) and according to each of the eight behaviors (Figure 2b).

The results showed higher scores for the sC/aSP condition than for the three other conditions in six/eight behaviors. For “emotional expression”, the score was high and very similar for the four conditions, and for “response to simple commands” it was low for the four conditions and not higher for the sC/aSP condition than for the three other conditions.

Wilcoxon signed-rank tests showed that the scores for the sC/aSP condition were significantly higher (68.1% of the max score) than those obtained for each of the three other conditions (mC/aSP: 40.6% of the max score, *p* = 0.044, *r* = 0.50; mC/nSP: 53.8% of the max score, *p* = 0.048, *r* = 0.49; and sC/nSP: 50% of the max score, *p* = 0.017, *r* = 0.60).

**Figure 3:**
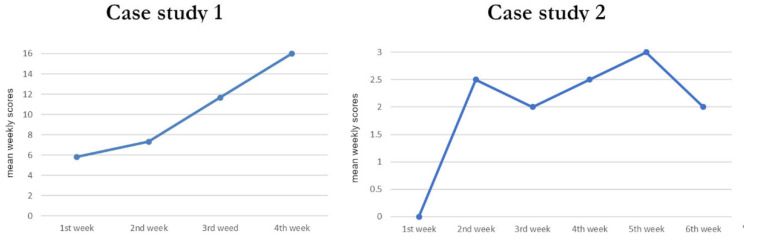
Time course of the average weekly behavioral score in the two case studies. The average score of several blocks performed in the same week is presented chronologically from week 1 to week 4 (case study 1) or week 6 (case study 2).

Regardless of the conditions, the mean weekly scores increased linearly (Figure 3, left) from week one (score = 5.83) to week four (score = 16). Wilcoxon signed-rank tests on time-corrected scores (i.e., after removing the linear increase in scores over time, thus potentially reflecting spontaneous improvement) showed significantly higher scores for the sC/aSP condition in comparison only to the sC/nSP condition (*p* = 0.015).

## 3. The Effect of Music Tempo on Subsequent Behavioral Assessment. Case Study Two

### 3.1. Method

#### 3.1.1. Patient

Mrs. O., 68 years old, was hospitalized following a brain hemorrhage during surgery for a left frontotemporal meningioma. After a coma phase (Glasgow Coma Scale: 3), she evolved towards an MCS. She had right hemi neglect and hemiplegia, and her left eye was permanently closed by the damage to the oculomotor nerve. The present experiment started 1095 days after the onset of coma. At the beginning of the present study, the patient was stable, nonsedated, had no indication of deafness (i.e., brainstem auditory-evoked potentials were present), and was in an MCS+ (16 points on the CRS-R: auditory function = 3 points, visual function = 5 points, motor function = 5 points, oromotor/verbal function = 1 point, communication = 0 points, and arousal = 2 points). At the end of the present study, the patient was still in an MCS+ (16 points). However, caregivers and the patient’s family noted changes in behavior a few days after the end of the experiment. They reported fewer periods of sleepiness during the day, more yes/no responses, more reactions to daily life, and less refusal of care.

The evaluations and experiments took place at the Physical Medicine and Rehabilitation Center in Saint Jean de Monts (France). The experiment was conducted in agreement with the guidelines of the Declaration of Helsinki. Written informed consent was signed by the patient’s relative.

#### 3.1.2. Materials

As in previous studies [17,18], we evaluated the effect of a context on a subset of CRS-R items, based on the possibility for improvement in Mrs. O. The objective was not to study the effects of context on a set of functions, such as those assessed in the CRS-R, but on the behaviors that could vary. Three items were selected at the beginning of the experiment from the complete CRS-R: “Movement to command”, “Object manipulation”, and “Communication”.

Three musical pieces of 3 min were created respecting the following conditions: music in major mode, without lyrics, and made up of only five distinct notes. Each musical piece had two verses, a chorus and a finale, and were simple and easy to listen to. All musical pieces were composed and played at a slow tempo (60 bpm) on the piano, and recorded in Audacity©. To isolate and specifically test the effects of tempo, we compared the same music at two different tempi: fast (180 bpm) and slow (60 bpm). The fast version was built from the slow version (it was played faster and recorded), so the music was repeated 3 times for the fast version (3 min). This manipulation allowed us to neutralize the effects of melody, rhythm, emotion, and familiarity, as well as holding other acoustic features constant across stimuli.

A white noise of 3 min was created in Audacity© for the control condition.

#### 3.1.3. Experimental Protocol

The study was conducted over six consecutive weeks, and consisted of 12 blocks of 30 min sessions (two blocks per week, three days apart, at the same time of day and by the same experimenter). A block corresponded to a quiet time of 15 min without any stimulation (silence), followed by three triplets (3 × 5 min).

**Figure 4:**
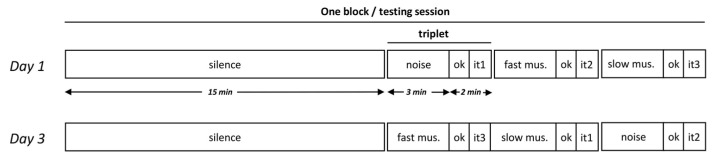
Experimental design for case study two. A block consisted of a 15 min period of silence, followed by 3 triplets, each of which consisted of one of the three types of sound (white noise, fast music, or slow music), the question “are you okay?” (ok), and one of the three selected CRS items (it). The three types of sounds and the three items (Movement to command, Object manipulation, and Communication) were used in one block, their combinations being mixed in a homogeneous and pseudo-random way between the blocks. Twelve blocks were presented throughout 6 weeks. The first two blocks are illustrated, corresponding to days 1 and 3.

A triplet (5 min) was made up of one of the three sounds (white noise, fast music, or slow music, 3 min), a question (“are you okay?”, see below, 1 min), and one of the three selected CRS-R items (“Movement to command”, “Object manipulation”, or “Communication”, 1 min). The three types of sounds and the three items were used in one block, their combinations being mixed in a homogeneous and pseudo-random way between the blocks (see Figure 4).

The sounds were delivered by a speaker placed two meters away from the patient.

#### 3.1.4. Behavioral Assessment

Following any sound (white noise, fast music, slow music) in a triplet, the question “are you okay?” was asked (Figure 4). The patient had to respond with the established yes/no code, i.e., by nodding (score from 0 point, i.e. no response, to 1 point for “yes” response, the “no” response was never observed).

Following the question “are you okay?”, one of the three selected items was administrated (and scored) according to the recommendations for the CRS-R (number of successes per item, score from 0 to 2 points).

#### 3.1.5. Data Analysis

Behavioral assessments for the 36 triplets (3 triplets within each of 12 blocks) were completed by one speech therapist who was blind to the condition (scoring was conducted from videos that were cut to disguise the experimental context). For each type of sound (white noise, fast music, or slow music), we calculated an average score by adding the score for the CRS-R item (“Movement to command”, “Object manipulation”, or “Communication”) and the score for the question. The data were presented according to the three types of sound (white noise, fast music, or slow music).

As previously done in single-case analyses (e.g., [18,42]), statistical analyses were performed on the 12 average scores of each type of sound using nonparametric tests (Friedman and Wilcoxon tests), and reported when significant (*p* < 0.05).

In order to observe a potential evolution of the scores over time, they were also averaged for each week, regardless of condition.

Finally, general behavior was rated, blind to the condition, during each triplet (sound + ok + item, i.e., for 5 min) by calculating the duration with eyes open and the number of reflexive or nonreflexive behaviors (see Table A2).

### 3.2. Results

**Figure 5:**
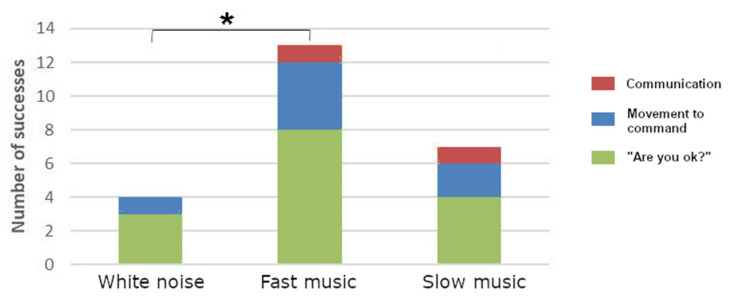
Number of successful responses to a question (“are you okay?”, in green) and to the CRS-R items (“Movement to command”, in blue, “Communication”, in red, and “Object manipulation” was never successfully completed), according to the experimental conditions (white noise, fast music, and slow music). * means *p* < 0.05.

The results showed a greater number of successful responses following fast music for one item (“Movement to command”, *n* = 4) and a response (“Are you okay?”, *n* = 8) as compared to slow music (“Movement to command”, *n* = 2; “Are you okay?”, *n* = 4), and for two items (“Movement to command”, *n* = 4; “Communication”, *n* = 1) and a response (“Are you okay?”, *n* = 8) as compared to white noise (“Movement to command”, *n* = 1; “Communication”, *n* = 0; and “Are you okay?”, *n* = 3) (see Figure 5). The item “Object manipulation” was never successfully completed by the patient.

The Wilcoxon tests performed on the average scores (12 for each type of sound) showed that the number of successes was significantly higher following fast music (*n* = 13) than following white noise (*n* = 4, *p* = 0.018).

Irrespective of condition, the mean weekly scores increased from week one (score = 0) to week six (score = 2), but not in a linear way (Figure 3, right).

Finally, for the general behavior in the full triplets, the average time with eyes open was higher when listening to fast music (92.9%) compared with slow music (80%) and white noise (74.6%). This difference was also found when the evaluation was conducted only during the presentation of a sound (93.8% in the fast music condition, 86.3% in the slow music condition, and 87% in the noise condition). We also observed more nonreflex behaviors in the fast music condition (*n* = 143) compared with the slow music condition (*n* = 105) and the control condition (*n* = 82). We did not observe any difference with the reflex behaviors (fast music: *n* = 35, slow music: *n* = 27, and noise: *n* = 38).

## 4. Discussion

As there is limited research on and official recommendations for the content of speech therapy sessions in DOC patients, the main objective of the present case studies was to explore whether the use of personally relevant and/or arousing stimuli could improve performance within a speech therapy session, which in turn could improve patient care. To this aim, we investigated (a) the effect of an autobiographical speech therapy session compared to a neutral speech therapy session on behavior within the session, and (b) the effects of a musical context, and its tempo, on a subsequent behavioral assessment.

Case study one showed the benefits, for the patient, of using autobiographical material during speech therapy sessions. We observed a significant increase in behavior and higher scores in almost all of the eight behaviors measured during speech therapy sessions using autobiographical material than during sessions using neutral material, as is currently implemented in speech therapy practice (Figure 2). Interestingly, the beneficial effect of autobiographical material remains even when the contribution of a possible spontaneous recovery was controlled for. These results are in accordance with previous neuroscientific studies that have shown beneficial effects of personally relevant stimuli on cognitive processing in DOC patients [12]. As suggested, this effect could be explained by overall cortical arousal and/or awareness enhancement following the activation of the autobiographical network [12]. Thus, the present case study encourages the use of relevant sensory stimuli that have familiar and emotional characteristics within speech therapy sessions.

Case study one showed higher scores when the autobiographical speech therapy session was preceded by a period of silence, compared to preferred music listening (Figure 2). No difference was observed between silent and preferred music contexts for neutral speech therapy sessions. This finding might mean that there was no beneficial effect of a preferred music context on behavior as compared to a silence context. However, this result does not corroborate the data in the literature regarding the importance of preferred music contexts on the cognitive and behavioral abilities of patients with a DOC [17,18,19]. Several hypotheses may explain this difference. The first hypothesis is associated with the fact that we chose a control condition without any stimulation (silence), whereas previous studies used a noise control condition. The differences could thus be explained by the fact that noise may have led to negative effects on patients’ behavioral abilities, which may not have been the case for the current silence control condition. The second hypothesis concerns the choice of music. The music pieces selected by the relative were not necessarily the most relevant for the patient, since it was not possible to ask the patient directly what their favorite music was. Moreover, in this case, the relative had chosen slow music, which may have had a relaxing effect (see below). The third hypothesis directly links to this relaxing effect since the duration of the preferred music was shorter in the previous studies (e.g., 30 s or 1 min) than in the present one (5 min). This difference in duration could lead to conflicting effects, with stimulating effects on brain functioning for short durations and global relaxing effects for longer durations. The relaxing or stimulating effects of music have often been observed (for example [19,43]) and could be explained by the fact that music influences both emotional and (neuro)physiological systems, placing it in an ideal position to regulate the psychological and cognitive states of patients between stimulation and relaxation [21]. These seemingly contradictory effects have also been observed in normal participants when the tempo of the music was changed, with slow music inducing relaxing effects and fast music tending to stimulate/awaken (see also [35]).

Case study two directly tested the relaxing/stimulating effect of music by investigating behavior after white noise, fast music, and slow music for the first time in a DOC patient. Behavioral outcomes were measured with three CRS-R items and the response to a simple question using a yes/no code. A significant enhancement of behavior was observed after fast music compared to white noise (Figure 5). These findings corroborate the hypothesis formulated by Gomez and Danuser, that the tempo of the music could influence the arousal of MCS patients [36]. From a qualitative point of view, the analysis of the patient’s behaviors during the sound/item pairings in case study two was in line with these conclusions. Indeed, the results suggest a longer duration of eye opening and a higher number of nonreflex behaviors when listening to fast music compared to slow music or white noise. It would have been interesting to record the physiological/vegetative variations (ECG, respiration, etc.) in order to observe whether changes in music tempo also affected vitals, including an increase in heart rate when listening to fast music and a decrease when listening to slow music [36,44]. Thus, our research encourages the use of musical stimuli with a fast tempo before a speech therapy session in DOC patients.

### Limitations

The current results should be interpreted with caution based on the small sample size, as they cannot necessarily be generalized to the DOC patient population. However, given the variability of lesions encountered in this patient population, a study with a larger number of patients would not necessarily help to generalize the results. To improve our statistical power, we rather opted for a longitudinal design across 4 to 6 weeks, with 6 h of testing per patient, which allowed for the repetition of conditions and statistics at the individual level. Such designs are essential for fluctuating patients with a DOC. Thus, the present study is a proof of concept that does not allow for firm conclusions to be drawn but informs future research into this area, as well as inform future speech therapy practices.

The current study did not investigate the long-term behavioral effects of personally relevant and/or arousing stimuli. The long-term effects of sensory stimulation programs, i.e., their rehabilitative potential, are less well-known than their short-term effects. According to Pape et al., the positive effects of familiar stimuli can be observed on long-term cognitive recovery [45]. Furthermore, the increased participation of DOC patients has also been observed with music therapy, suggesting enhanced motivation with auditory stimulation [46,47]. However, it is difficult to draw firm conclusions from these studies as they did not use quantitative measures and were missing control conditions/groups (for a review, see [8]). In the present case study one, an overall increase in weekly scores was observed across the 4 weeks (Figure 3). Although the CRS-R score was the same at the beginning and at the end of the experiment (13 points), caregivers reported more stereotypies, yes/no responses, and communicative gestures at the end of the experiment. In the present case study two, an increase in weekly score was also observed, but more marginally (Figure 3). Although the CRS-R score was the same at the beginning and at the end of the experiment (16 points), caregivers and the patient’s family reported fewer periods of sleepiness during the day, more yes/no responses, more reactions to daily life, and less refusal of care at the end of the experiment. Only a future study comparing the recovery of patients who received autobiographical stimuli with that of patients who received neutral stimuli, or using a withdrawal design (i.e., alternating periods of baseline with periods of treatment, as in [48]), could answer the question of the long-term benefits of autobiographical stimulation.

## 5. Conclusions

The aim of the two present case studies was to investigate whether behavior can be improved in speech therapy practice through the selection of meaningful and/or arousing contexts and stimuli. The role of a speech–language pathologist with DOC patients is to identify signs of arousal and awareness in order to stimulate the emergence of cognitive and communicative skills that allow patients to interact with the world. A speech therapist’s main objective is to solicit and support the emergence of consciousness and the development of functional verbal or nonverbal communication. A speech therapist also intervenes in the evaluation and rehabilitation of swallowing and phonation disorders. Nevertheless, scientific research and official recommendations regarding a speech therapist’s assessment, diagnosis, and treatment of patients with DOC are limited. The current results highlight the value of autobiographical stimuli and fast-tempo music to improve the short-term assessment of DOC patients’ behavior. If these findings are validated within larger samples, these types of stimuli could be used within speech therapy practice to better assess patients during speech therapy and, perhaps, in rehabilitation programs to promote recovery.

## Data Availability

Due to the clinical nature of patient data and institutional restriction, data are available from the corresponding author upon request.

## Appendix A

**Table A1:** Behavioral Reactions Observed During the Speech Therapy Session.

	Scoring/Items	Qualitative Observations
PRECURSORS TO COMMUNICATION	**1.** **Eye Contact** □ 0 points: No eye contact □ 1 point: Fluctuating eye contact with stimulation □ 2 points: Permanent eye contact with stimulation □ 3 points: Permanent and spontaneous eye contact **2.** **Attentional Abilities in Interaction (Eye Tracking, Posture, Joint attention…)** □ 0 points: No attention to the speech therapist, despite solicitations □ 1 point: Fluctuating attention regardless of solicitations □ 2 points: Fluctuating attention but interactions maintained by the solicitations □ 3 points: Permanent attention allowing the maintenance of the interaction **3.** **Expression of Affects (Gestures, Vocal Expressions, Grimaces, Tears, Smiles, etc.)** □ 0 points: No emotional reaction □ 1 point: Difficult interpretable emotional reactions □ 2 points: Clearly interpretable emotional reactions	
	Score: /8	
ORAL/PERIORAL	**4.** **Reactions to Oral and Perioral Solicitations** □ 0 points: No reactions/archaic reflex □ 1 point: Automatic tongue movements/mouth opening on contact +/− swallowing □ 2 points: Mouth opening on approach with +/− closing and +/− swallowing □ 3 points: Spontaneous mouth opening and closing followed by swallowing	
	Score: /3	
COMMUNICATION SKILLS	**5.** **Responses (verbal, motor) to simple commands** □ 0 points: No response □ 1 point: Fluctuating responses □ 2 points: Permanent responses **6.** **Yes/No code** □ 0 points: No yes/no code □ 1 point: Undifferentiated yes/no or single response □ 2 points: Differentiated but fluctuating yes/no □ 3 points: Differentiated and permanent yes/no **7.** **Vocal or verbal reactions** □ 0 points: No vocal reaction/Involuntary or reflex sounds □ 1 point: Voluntary non-meaningful or non-intelligible sounds □ 2 points: Voluntary and meaningful sounds **8.** **Motor manifestations** □ 0 points: No movement except reflexes or stereotypes □ 1 point: Self-directed movements □ 2 points: Conventional movements □ 3 points: Gestures for communicative purposes (comprehension/expression)	Yes/no code: Spontaneous? On solicitation? Reliable?
	Score: /10	
	**OVERALL SCORE:** /21	

Other behaviors that may be observed (qualitative):

□ Appeasement/sleepiness
□ Agitation
□ Opening the eyes when the door is opened
□ Opening of the eyes at the beginning of the music
□ Orientation of the eyes towards the speakers
□ Emotional reactions (smiling, crying, laughing etc.)
□ Looking through the door
□ Others: …………………………………………………………………..

## Appendix B

**Table A2:** Behavioral observation.

**Reflex Behaviors**	**Number of Achievement** **Observations**
Chewing	
Swallowing	
Yawning	
Coughing	
**Non-reflex behaviors**	**Number of achievement** **Observations**
Moving the head	
Changing of eyes orientation	
Changing in position	
Scratching	
Touching a part of the body	
Acting on an element of the environment (tracheostomy cannula, saturometer, wheelchair, etc.)	
Nodding	

## References

[1] Multi-Society Task Force on PVS (1994). Medical aspects of the persistent vegetative state. N. Engl. J. Med..

[2] Giacino J.T., Ashwal S., Childs N., Cranford R., Jennett B., Katz D.I., Kelly J.H., Rosenberg J., Whyte J., Zafonte R.D. (2002). The minimally conscious state definition and diagnostic criteria. Neurology.

[3] Bruno M.-A., Vanhaudenhuyse A., Thibaut A., Moonen G., Laureys S. (2011). From unresponsive wakefulness to minimally conscious PLUS and functional locked-in syndromes: Recent advances in our understanding of disorders of consciousness. J. Neurol..

[4] Cassol H., Aubinet C., Thibaut A., Wannez S., Martial C., Martens G., Laureys S. (2018). Diagnostic, pronostic et traitements des troubles de la conscience. NPG Neurol.-Psychiatr.-Gériatrie.

[5] (2019). General Nomenclature of Speech Therapy Procedures, French Official Journal. https://www.legifrance.gouv.fr/jorf/jo/2019/07/02/0151.

[6] Mortensen J., Jensen D., Kjaersgaard A. (2016). A validation study of the Facial-Oral Tract Therapy Swallowing Assessment of Saliva. Clin. Rehabil..

[7] Mélotte E., Belorgeot M., Herr R., Simon J., Kaux J.F., Laureys S., Sanz L.R., Lagier A., Morsomme D., Pellas F. (2021). The Development and Validation of the SWADOC: A Study Protocol for a Multicenter Prospective Cohort Study. Front. Neurol..

[8] Lancioni G.E., Bosco A., Belardinelli M.O., Singh N.N., O’Reilly M.F., Sigafoos J. (2010). An overview of intervention options for promoting adaptive behavior of persons with acquired brain injury and minimally conscious state. Res. Dev. Disabil..

[9] Di Stefano C., Cortesi A., Masotti S., Simoncini L., Piperno R. (2012). Increased behavioural responsiveness with complex stimulation in VS and MCS: Preliminary results. Brain Inj..

[10] Meyer M.J., Megyesi J., Meythaler J., Murie-Fernandez M., Aubut J.A., Foley N., Salter K., Bayley M., Marshall S., Teasell R. (2010). Acute management of acquired brain injury Part III: An evidence-based review of interventions used to promote arousal from coma. Brain Inj..

[11] Woisard V., Puech M. (2013). La Réhabilitation de la Déglutition Chez L’adulte.

[12] Perrin F., Castro M., Tillmann B., Luauté J. (2015). Promoting the use of personally relevant stimuli for investigating patients with disorders of consciousness. Front. Psychol..

[13] Bekinschtein T., Niklison J., Sigman L., Manes F., Leiguarda R., Armony J., Owen A., Carpintiero S., Olmos L. (2014). Emotion processing in the minimally conscious state. J. Neurol. Neurosurg. Psychiatry.

[14] Sharon H., Pasternak Y., Ben Simon E., Gruberger M., Giladi N., Krimchanski B.Z., Hassin D., Hendler T. (2013). Emotional Processing of Personally Familiar Faces in the Vegetative State. PLoS ONE.

[15] Signorino M., D’Acunto S., Cercaci S., Pietropaoli P., Angeleri F. (1997). The P300 in traumatic coma: Conditioning of the odd-ball paradigm. J. Psychophysiol..

[16] Perrin F., Schnakers C., Schabus M., Degueldre C., Goldman S., Brédart S., Faymonville M.-E., Lamy M., Moonen G., Luxen A. (2006). Brain response to one’s own name in vegetative state, minimally conscious state, and locked-in syndrome. Arch. Neurol..

[17] Verger J., Ruiz S., Tillmann B., Ben Romdhane M., De Quelen M., Castro M., Tell L., Luauté J., Perrin F. (2014). Effets bénéfiques de la musique préférée sur les capacités cognitives des patients en état de conscience minimale. Rev. Neurol..

[18] Heine L., Tillmann B., Hauet M., Juliat A., Dubois A., Laureys S., Kandel M., Plailly J., Luauté J., Perrin F. (2017). Effects of preference and sensory modality on behavioural reaction in patients with disorders of consciousness. Brain Inj..

[19] Castro M., Tillmann B., Luauté J., Corneyllie A., Dailler F., André-Obadia N., Perrin F. (2015). Boosting cognition with music in patients with disorders of consciousness. Neurorehabilit. Neural Repair.

[20] Altenmüller E., McPherson G.E., Gruhn W., Rauscher F.H. (2007). Motor Learning and Instrumental Training. Neurosciences in Music Pedagogy.

[21] Moussard A., Rochette F., Bigand E. (2012). La musique comme outil de stimulation cognitive. L’année Psychol..

[22] Wan C.Y., Schlaug G. (2010). Music making as a tool for promoting brain plasticity across the life span. Neurosci. A Rev. J. Bringing Neurobiol. Neurol. Psychiatry.

[23] Altenmüller E., Schlaug G. (2015). Apollo’s gift: New aspects of neurologic music therapy. Prog. Brain Res..

[24] Fukui H., Toyoshima K. (2008). Music facilitate the neurogenesis, regeneration and repair of neurons. Med. Hypotheses.

[25] Grimm T., Kreutz G. (2018). Music interventions in disorders of consciousness (DOC)—A systematic review. Brain Inj..

[26] Magee W.L. (2005). Music therapy with patients in low awareness states: Approaches to assessment and treatment in multidisciplinary care. Neuropsychol. Rehabil..

[27] Magee W.L. (2007). Music as a diagnostic tool in low awareness states: Considering limbic responses. Brain Inj..

[28] Baker F.A. (2000). Modifying the melodic intonation therapy program for adults with severe non-fluent aphasia. Music Ther. Perspect..

[29] Cohen N.S. (1988). The use of superimposed rhythm to decrease the rate of speech in a braindamaged adolescent. J. Music Ther..

[30] Cohen N.S. (1992). The effect of singing instruction on the speech production of neurologically impaired persons. J. Music Ther..

[31] Cohen N.S., Masse R. (1993). The Application of Singing and Rhythmic Instruction as a Therapeutic Intervention for Persons with Neurogenic Communication Disorders. J. Music Ther..

[32] Pilon M.A., McIntosh K.W., Thaut M.H. (1998). Auditory vs. visual speech timing cues as external rate control to enhance verbal intelligibility in mixed spastic-ataxic dysarthric speakers: A pilot study. Brain Inj..

[33] Heine L., Castro M., Martial C., Tillmann B., Laureys S., Perrin F. (2015). Exploration of Functional Connectivity During Preferred Music Stimulation in Patients with Disorders of Consciousness. Front. Psychol..

[34] Carrière M., Larroque S.K., Martial C., Bahri M.A., Aubinet C., Perrin F., Laureys S., Heine L. (2020). An Echo of Consciousness: Brain Function During Preferred Music. Brain Connect..

[35] Bernardi L., Porta C., Sleight P. (2006). Cardiovascular, cerebrovascular, and respiratory changes induced by different types of music in musicians and non-musicians: The importance of silence. Heart.

[36] Gomez P., Danuser B. (2007). Relationships Between Musical Structure and Psychophysiological Measures of Emotion. Emotion.

[37] Okada K., Kurita A., Takase B., Otsuka T., Kodani E., Kusama Y., Atarashi H., Mizuno K. (2009). Effects of music therapy on autonomic nervous system activity, incidence of heart failure events, and plasma cytokine and catecholamine levels in elderly patients with cerebrovascular disease and dementia. Int. Heart J..

[38] Giacino J.T., Kalmar K., Whyte J. (2004). The JFK Coma Recovery Scale-Revised: Measurement characteristics and diagnostic utility. Arch. Phys. Med. Rehabil..

[39] Shiel A., Horn S.A., Wilson B.A., Watson M.J., Campbell M.J., McLellan D.L. (2000). The Wessex Head Injury Matrix (WHIM) main scale: A preliminary report on a scale to assess and monitor patient recovery after severe head injury. Clin. Rehabil..

[40] Garin J., Reina M. (2013). Création et Validation d’une Echelle d’Évaluation de la Communication pour des Patients en Phase d’Éveil de Coma (ECEC). http://docnum.univ-lorraine.fr/public/BUMED_MORT_2013_GARIN_JULIE_REINA_MARGOT.pdf.

[41] Rousseaux M., Delacourt A., Wyrzykowski N., Lefeuvre M. (2001). TLC: Test Lillois de communication. Communication Test.

[42] Soto D., Funes M.I., Guzmán-García A., Warbrick T., Rotshtein P., Humphreys G.W. (2009). Pleasant music overcomes the loss of awareness in patients with visual neglect. Proc. Natl. Acad. Sci. USA.

[43] Khalfa S., Bella S.D., Roy M., Peretz I., Lupien S.J. (2003). Effects of relaxing music on salivary cortisol level after psychological stress. Ann. N. Y. Acad. Sci..

[44] Magee W.L. (2018). Music in the diagnosis, treatment and prognosis of people with prolonged disorders of consciousness. Neuropsychol. Rehabil..

[45] Mollakazemi M.J., Biswal D., Elayi S.C., Thyagarajan S., Evans J. (2019). Synchronization of Autonomic and Cerebral Rhythms During Listening to Music: Effects of Tempo and Cognition of Songs. Physiol. Res..

[46] Pape T.L.-B., Rosenow J.M., Steiner M., Parrish T., Guernon A., Harton B., Patil V., Bhaumik D.K., McNamee S., Walker M. (2015). Placebo-controlled trial of familiar auditory sensory training for acute severe traumatic brain injury: A preliminary report. Neurorehabilit. Neural Repair.

[47] Formisano R., Vinicola V., Penta F., Matteis M., Brunelli S., Weckel J.W. (2001). Active music therapy in the rehabilitation of severe brain injured patients during coma recovery. Ann. Dell’istituto Super. Sanita.

[48] Cheng L., Cortese D., Monti M.M., Wang F., Riganello F., Arcuri F., Di H., Schnakers C. (2018). Do Sensory Stimulation Programs Have an Impact on Consciousness Recovery?. Front. Neurol..

